# Fructose metabolism as an adaptive survival axis in pancreatic cancer: mechanistic insights and therapeutic implications

**DOI:** 10.3389/fmed.2026.1794440

**Published:** 2026-04-20

**Authors:** Yuan Zhang, Ye Zhang

**Affiliations:** Department of General Surgery, The Affiliated Wuxi People’s Hospital of Nanjing Medical University, Wuxi, China

**Keywords:** AMPK/mTORC1 pathway, fructose metabolism, metabolic reprogramming, pancreatic cancer, stress granules

## Abstract

While previous research has established fructose as an alternative carbon source for tumors and implicated it in the metabolic reprogramming of pancreatic cancer, a comprehensive understanding of its coordinated regulation through exogenous uptake and endogenous synthesis, and its coupling with stress adaptation mechanisms like stress granules (SGs) to form an “adaptive survival axis,” remains incomplete. This study employed a mechanism-oriented narrative review approach to integrate existing evidence on the regulatory mechanisms of fructose metabolism and stress response signaling in pancreatic ductal adenocarcinoma (PDAC), based on literature identified from PubMed, Web of Science, and CNKI databases. Our findings indicate that fructose is taken up via GLUT5 and endogenously synthesized through the Pentose Phosphate Pathway (PPP). It subsequently modulates redox homeostasis and autophagy via the pentose phosphate pathway, lipid synthesis, and the AMPK/mTORC1 signaling pathway. Concurrently, fructose induces eIF2α phosphorylation and the phase separation of RNA-binding proteins to form SGs. These SGs act as a metabolic-signaling coupling platform, synergizing with the KRAS-NUPR1 pathway to enhance tumor stress tolerance. By integrating these pathways, this study innovatively proposes a framework of the “fructose metabolism-driven adaptive survival axis” in PDAC, positing fructose metabolism as a critical adaptive survival axis that combines metabolic reprogramming with stress tolerance. This provides a theoretical basis for developing synthetic lethality strategies targeting “fructose uptake/metabolic enzymes + blockade of SG formation” and precision nutritional interventions. Consequently, this research not only advances our understanding of pancreatic cancer metabolism but also reveals therapeutic opportunities involving metabolic targeting, stress granule modulation, and precision nutritional intervention.

## 1 Introduction

Pancreatic cancer is a fatal disease (1), ranking seventh among cancer-related causes of death globally (2). Pancreatic ductal adenocarcinoma (PDAC) accounts for as much as 90% of cases (3), with a 5-year survival rate of less than 10% following diagnosis (4). Emerging research reveals a consistent upward trend in pancreatic cancer incidence among younger females (5), highlighting the pressing necessity to decipher its underlying pathogenic mechanisms. The malignant characteristics of PDAC stem from uncontrolled cell proliferation and potent invasive capabilities (6), both of which rely on extensive metabolic reprogramming. This adaptation enables cancer cells to survive and proliferate within the hypoxic, nutrient-depleted environment unique to pancreatic tissue (7).

While glucose metabolism has historically been central to tumor bioenergetics research, forming the cornerstone of classic theories on metabolic reprogramming in cancer (8), in the glucose-deficient tumor microenvironment, fructose—a monosaccharide widely found in natural foods and industrialized food additives (9)—has been identified as a critical alternative carbon source for tumor cells. Its metabolic process exhibits regulatory characteristics distinctly different from those of glucose. Unlike glucose, which is broadly taken up via multiple glucose transporters (GLUTs) and phosphorylated by hexokinase (HK) to enter the classic glycolytic pathway, fructose primarily enters cells through the fructose-specific transporter GLUT5, encoded by the solute carrier family 2 member 5 (SLC2A5) gene (10). Its metabolic flux bypasses phosphofructokinase 1 (PFK1), a key rate-limiting step in glycolysis, forming a regulatory pathway independent of glucose metabolism. Existing studies have confirmed that fructose can serve as an alternative energy source for tumor initiation and progression, particularly in glucose-depleted microenvironments. Various tumor cells, including those in acute myeloid leukemia, lung cancer, and colorectal cancer (11, 12), enhance their fructose utilization capacity by upregulating GLUT5 expression, thereby maintaining their malignant proliferative phenotype. However, in contrast to the emphasis on glucose dependency in classic tumor metabolism theories, pancreatic ductal adenocarcinoma (PDAC) possesses a unique tumor microenvironment characterized by hypovascularity, dense stroma, and extreme nutrient scarcity (13). Consequently, therapeutic strategies solely targeting glucose metabolism have shown minimal clinical efficacy. Recent evidence suggests that fructose metabolism in PDAC is not merely a supplementary pathway to glucose metabolism but constitutes an independent adaptive survival axis that drives tumor adaptation to nutritional stress (14). Currently, A systematic review and thorough elucidation of the specific regulatory mechanisms, upstream and downstream signaling networks, and clinical translational value of fructose metabolism as a core strategic “survival axis” in PDAC remain lacking. Therefore, this review proposes a conceptual framework entitled the “fructose metabolism-driven adaptive survival axis,” which systematically integrates the core molecular mechanisms of metabolic input, metabolic reprogramming, signal regulation and stress adaptation in pancreatic ductal adenocarcinoma.

To achieve this goal, in this mechanism-oriented narrative review, we systematically retrieved evidence from PubMed, Web of Science Core Collection, and China National Knowledge Infrastructure (CNKI) to synthesize current advances in fructose uptake, metabolism, and signaling regulation within pancreatic ductal adenocarcinoma. We focus on how fructose, via GLUT5-mediated transport and both exogenous and endogenous metabolic pathways, rewires pentose phosphate flux, lipid biosynthesis, and Adenosine Monophosphate-Activated Protein Kinase (AMPK) -inositol signaling pathways. This coordination underpins redox homeostasis, autophagy suppression, and stress adaptation within nutritionally deprived pancreatic tumors. Through the integration of these metabolic, signaling, and stress-responsive pathways, we put forward the notion that fructose metabolism serves as a pivotal adaptive survival axis in pancreatic cancer. This unified framework not only deepens our understanding of tumor metabolic plasticity but also provides actionable opportunities for metabolic-targeted therapies, patient stratification, and precision nutritional interventions, thereby expanding the frontiers of pancreatic cancer prevention and treatment research.

## 2 Methods

### 2.1 Literature search

A literature search was conducted using PubMed, Web of Science Core Collection, and China National Knowledge Infrastructure (CNKI), covering publications from database inception to December 2025. The search aimed to identify relevant studies addressing fructose metabolism and its role in pancreatic cancer. Search terms included “fructose metabolism,” “pancreatic cancer,” “pancreatic ductal adenocarcinoma,” “GLUT5,” “SLC2A5,” “hexokinase,” “polyol pathway,” “metabolic reprogramming,” and “tumor metabolism,” combined using Boolean operators. In addition, reference lists of relevant articles were manually screened to identify further studies.

### 2.2 Literature selection and synthesis

Studies were selected based on their relevance to the topic, with particular emphasis on research investigating molecular mechanisms, metabolic pathways, and tumor progression associated with fructose metabolism in pancreatic cancer. Priority was given to peer-reviewed original research, reviews, and translational studies. This review follows a narrative approach and does not adhere to a formal systematic review protocol (e.g., PRISMA). Therefore, the literature identification and selection processes were not designed to be exhaustive or fully reproducible.

Due to substantial heterogeneity in study designs and experimental models, a qualitative synthesis was conducted. This approach is consistent with mechanism-oriented narrative reviews aiming to provide conceptual integration. The analysis is structured around three key themes: fructose source regulation, metabolic reprogramming, and downstream signaling responses.

### 2.3 Ethical statement

This review is based on previously published research and does not involve any new human or animal experiments. Consequently, ethical approval and informed consent are not required.

## 3 Regulation of pancreatic cancer cell proliferation by fructose metabolism

### 3.1 Metabolic input layer: regulation of exogenous and endogenous fructose expression

Existing evidence supports the classification of dietary fructose into two distinct types, differentiated by their sources and modes of ingestion: naturally occurring fructose (e.g., in fruits and root vegetables) and industrially added fructose (e.g., in high-fructose corn syrup and sucrose). Although the fructose monomers in both categories are chemically identical, the differences in their physiological metabolic effects are not inherent to fructose itself. Instead, they are determined by the food matrix carrying the fructose, its form of presence, and the accompanying dietary components (15). Significant disparities exist along the complete physiological pathway of “intake pattern—food matrix—absorption kinetics—metabolic response” (Table 1). Fructose in natural foods predominantly exists as sucrose, linked to glucose via an α-1,2 glycosidic bond, and is often found alongside dietary fiber, pectin, and other components within whole fruits and root vegetables. The dietary fiber in these foods can slow gastric emptying, reduce localized fructose concentrations in the intestine, and modulate fructose absorption kinetics, leading to a relatively slower and more controlled absorption and metabolism of fructose (15). Conversely, industrially added fructose is frequently used as a food additive in forms such as free fructose, high-fructose corn syrup, or refined sucrose, and is widely present in processed foods like sugar-sweetened beverages and snack items. These lack the buffering effect of dietary fiber or other accompanying dietary components. Such free fructose can be rapidly absorbed by the intestine, entering the body’s metabolic pathways in large quantities within a short period, easily overwhelming the body’s fructose metabolic buffering capacity and leading to metabolic overload (9).

**TABLE 1:** Differences in exogenous and endogenous expression regulation of fructose.

No.	Source	Representation	Characteristics
1	Natural	Fruits, root vegetables, etc.	Often co-exists with dietary fiber, slow absorption rate.
2	Added sugars	Corn syrup, sucrose, agave syrup, etc.	Widely present in food products, leading to metabolic overload.

The regulation of exogenous fructose centers on three key pathways: intestinal absorption, hepatic uptake, and metabolic activation. Each pathway is co-regulated by specific molecules and transcription factors. (1) In the intestinal absorption pathway, GLUT5 (SLC2A5) serves as the fructose-specific transporter. Its expression is regulated by the transcription factor ChREBP and can be up-regulated by dietary fructose. At high fructose concentrations, GLUT2 (SLC2A2) participates in transport synergistically, forming an absorption redundancy mechanism with GLUT5 (16). At physiological doses, approximately 90% of fructose is metabolized within intestinal epithelial cells during first-pass metabolism. Following phosphorylation by ketohexokinase (KHK), the majority is converted into organic molecules such as glucose, lactate, and citrate, with only a small amount of free fructose released into the portal circulation. Conversely, with high fructose intake, intestinal fructose transport and metabolic capacity become rapidly saturated. Over 70% of free fructose can then directly enter the portal circulation, significantly increasing the metabolic load on the liver (17). This dose-dependent difference in intestinal handling is the critical initiating step that leads to the divergent metabolic effects observed with high versus low fructose diets. (2) In the hepatic uptake phase, free fructose from the portal circulation is primarily internalized by hepatocytes. GLUT8 (SLC2A8) is not the sole transporter mediating fructose influx into the liver. Under physiological conditions, hepatocytes predominantly transport fructose across their membranes via GLUT2. Conversely, GLUT8, functioning as a high-affinity fructose transporter, is primarily involved in hepatic fructose uptake during conditions of high fructose exposure (18). Its expression is specifically regulated by the hepatic fructose sensor angiopoietin-like protein 3 (ANGPTL3). Fructose exposure can elevate circulating ANGPTL3 levels, which in turn upregulates hepatocyte GLUT8 expression via a paracrine pathway, thereby amplifying the liver’s fructose uptake capacity (16, 19). Significant differential effects are also observed at the hepatic level between low- and high-fructose diets. With low fructose intake, the small amount of fructose entering the liver is rapidly metabolized for glycogen synthesis or energy provision. In contrast, high fructose diets deliver substantial portal fructose, which rapidly activates the highly active KHK-C isoform within hepatocytes. This bypasses the rate-limiting steps of glycolysis, continuously generating metabolic intermediates and driving hepatic *de novo* lipogenesis and metabolic dysregulation (20). (3) In the metabolic initiation phase, hexokinase (HK) and ketohexokinase (KHK) represent two distinct classes of phosphorylating kinases with entirely different functions. HK is a key rate-limiting enzyme in glycolysis, preferentially phosphorylating glucose. Its activity is strongly negatively regulated by product feedback; although it can non-specifically phosphorylate fructose, its substrate affinity for glucose is far greater than for fructose, contributing minimally to fructose metabolism under physiological conditions. KHK, conversely, is the specific rate-limiting enzyme for fructose metabolism, uniquely catalyzing the phosphorylation of fructose to fructose-1-phosphate. Currently, no classical negative feedback regulatory mechanism for KHK has been identified; the flux of fructose metabolism is solely determined by the expression level of KHK itself (9). KHK primarily phosphorylates the 1-position of the hexose ring, whereas HK phosphorylates the 6-position, generating fructose-6-phosphate (F6P). F6P can enter the PPP. Furthermore, fructose can be converted to F6P via gluconeogenesis, subsequently transforming into glucose-6-phosphate (G6P), which enters the PPP as a substrate. Its downstream products enter the non-oxidative branch of the PPP via transketolase (TKT) and transaldolase (TAL). The KHK gene undergoes alternative splicing to produce two functionally divergent isoforms: KHK-A and KHK-C. KHK-C exhibits extremely high affinity for fructose and is the functional subtype responsible for fructose metabolism. Its transcription is specifically regulated by carbohydrate-responsive element-binding protein (ChREBP), directly dictating the metabolic fate of cellular fructose. KHK-A has a very low affinity for fructose, is broadly expressed at low levels in systemic tissues, and plays no core regulatory role in fructose metabolism (18). It is noteworthy that this review focuses on the pivotal role of KHK-C in pancreatic ductal adenocarcinoma (PDAC), rather than KHK-A. This focus is primarily based on the specific molecular characteristics and metabolic phenotypes of PDAC tumor tissues and cells. Firstly, PDAC tumor tissues exhibit specific high expression of KHK-C, with extremely low expression of KHK-A. Moreover, high KHK-C expression is significantly correlated with poor patient prognosis. Secondly, in the glucose-deprived tumor microenvironment of PDAC, only the high-affinity KHK-C can efficiently initiate fructose metabolism, providing an alternative carbon source for tumor cells. Finally, functional experiments have confirmed that inhibiting KHK-C completely abrogates fructose-mediated PDAC malignant phenotypes, whereas manipulating KHK-A has no significant effect (21).

**FIGURE 1:**
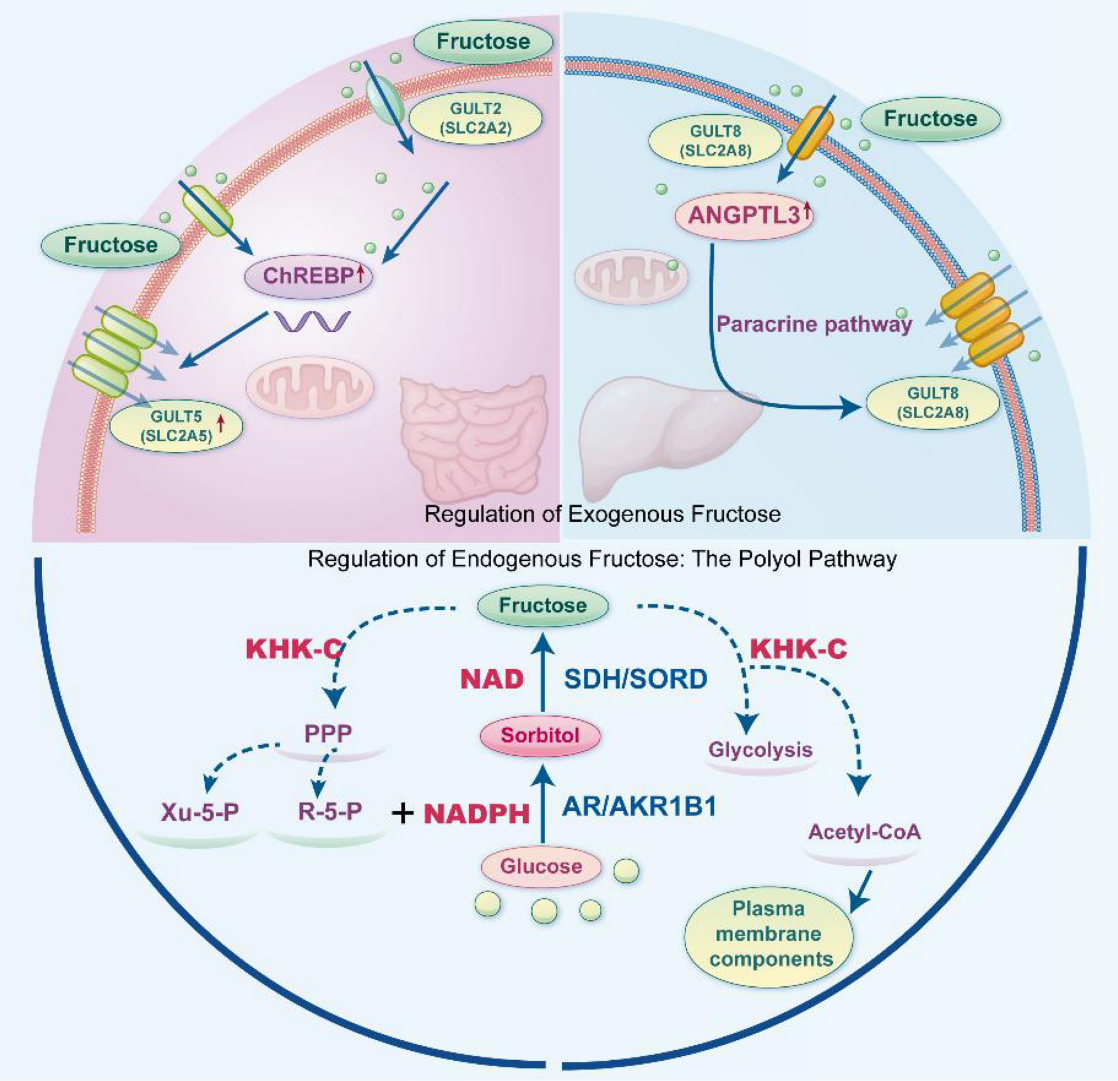
Schematic representation of the regulation of exogenous and endogenous fructose in cellular metabolism. Exogenous fructose uptake by intestinal epithelial cells occurs via GLUT5/GLUT2, with ChREBP transcriptionally upregulating GLUT5 to enhance this process. Hepatic-derived ANGPTL3 promotes GLUT8 expression through a paracrine mechanism, further augmenting fructose transport efficiency. Endogenous fructose is synthesized from glucose via the polyol pathway: AR/AKR1B1 catalyzes glucose to sorbitol, and SDH/SORD subsequently converts sorbitol to fructose. The resulting fructose is shunted by KHK-C regulation, either entering the pentose phosphate pathway (PPP) to generate ribose-5-phosphate and xylulose-5-phosphate, or entering glycolysis to produce acetyl-CoA for *de novo* membrane component synthesis.

Human endogenous fructose synthesis relies solely on the polyol pathway, which converts glucose to fructose via two enzymatic steps: AR/AKR1B1 catalyzes glucose to sorbitol (NADPH-dependent, rate-limiting step) (22); and sorbitol dehydrogenase (SDH/SORD) catalyzes the conversion of sorbitol to fructose (requiring NAD as a coenzyme). The resulting fructose can directly enter cellular metabolic cycles (23). These mechanisms collectively form a highly inducible fructose uptake and activation module. This module sensitizes pancreatic tumors to dietary fructose availability, serving as a key input foundation for the “fructose metabolism-driven adaptive survival axis” (Figure 1).

The core regulation of endogenous fructose synthesis hinges on the synergistic interplay between key enzyme activity and metabolic environment. Tumor tissues exhibit specific abnormal activation characteristics: (1) Key enzyme regulation: Tumor cells show significantly upregulated AR (AKR1B1) expression, whose activity is induced by hypoxia and the Warburg effect (24). Concurrently elevated SDH expression accelerates endogenous fructose production (25); (2) Metabolic reprogramming in the tumor microenvironment (e.g., hypoxia, high glucose) upregulates the pentose phosphate pathway (PPP), thereby increasing the NADPH/NADP^+^ ratio. A high ratio significantly promotes AR-catalyzed reactions, further enhancing endogenous fructose synthesis flux (22); (3) Tumor-specific pathways: AR-mediated endogenous fructose metabolism directly promotes tumor cell migration and metastasis by activating the RhoA-ROCK2 pathway. Supplementing exogenous fructose reverses the reduced metastatic capacity caused by AR deficiency (24). Endogenous fructose provides tumor cells with additional energy substrates, enhancing metabolic flexibility to support rapid proliferation. Simultaneously, by regulating migration-related pathways, it promotes tumor invasion and distant metastasis, serving as a key driver of malignant tumor phenotypes. This endogenous fructose generation pathway enables tumor cells to circumvent extracellular nutrient limitations and maintain metabolic plasticity. Together with exogenous fructose uptake, it constitutes a dual material supply assurance for the “fructose metabolism-driven adaptive survival axis.”

### 3.2 Metabolic reprogramming layer: pentose phosphate pathway, lipid synthesis

**FIGURE 2:**
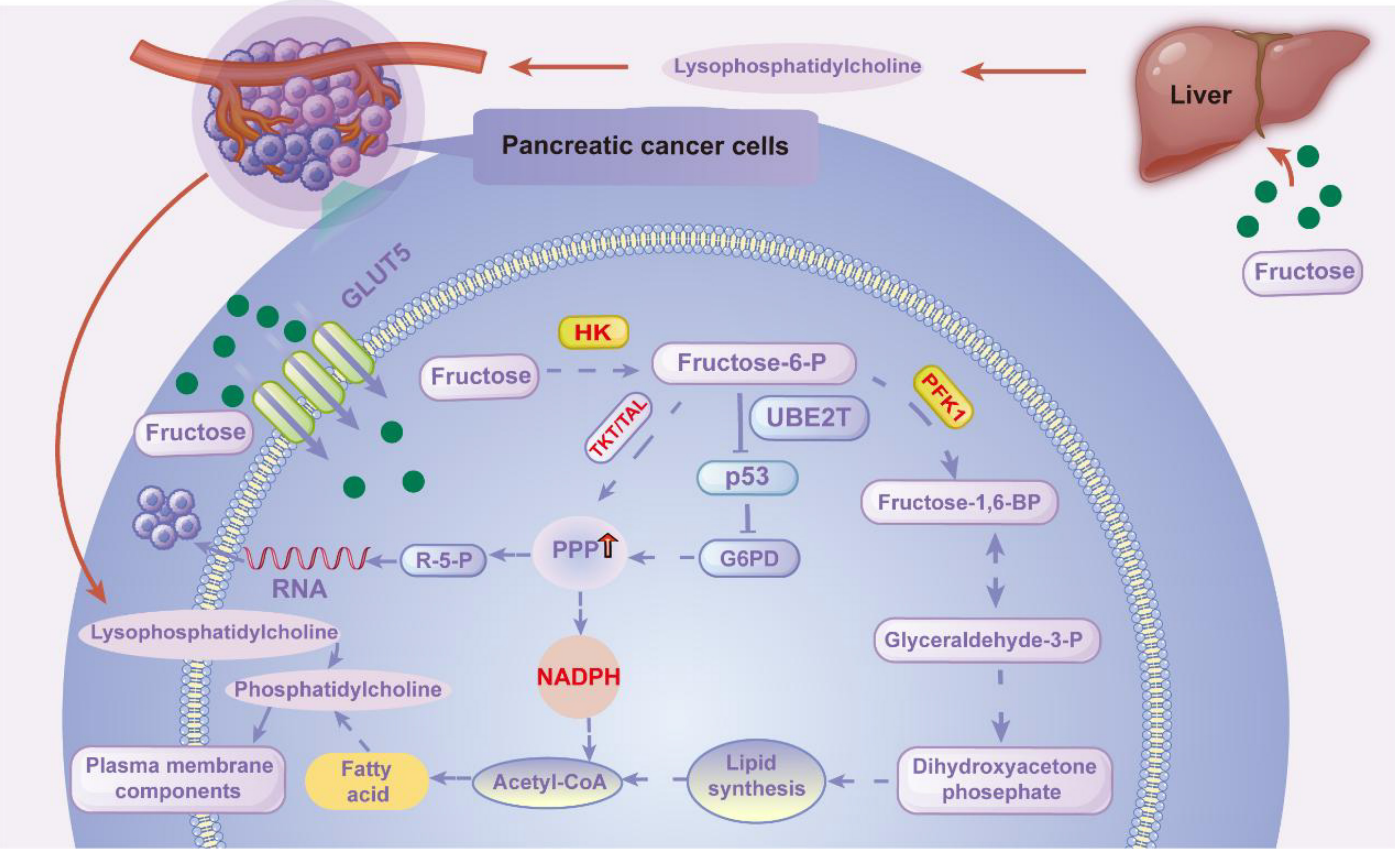
Schematic diagram illustrating the mechanism by which fructose metabolism drives adaptive survival in pancreatic ductal adenocarcinoma (PDAC). Exogenous fructose is taken up by PDAC cells via GLUT5, subsequently metabolized by ketohexokinase (KHK) and glucose-6-phosphate dehydrogenase (G6PD). This process activates the pentose phosphate pathway (PPP), promoting NADPH generation for redox homeostasis, while supporting *de novo* lipogenesis via acetyl-CoA for membrane component synthesis, such as phosphatidylcholine. Concurrently, fructose induces phase separation of RNA-binding proteins into stress granules (SGs); SGs serve as a platform coupling metabolic flux to KRAS-NUPR1 signaling, thereby enhancing PDAC cell stress tolerance. Targeting key nodes along this axis (GLUT5, KHK/G6PD, SG regulators) may enable a synthetic lethality therapeutic approach for PDAC. GLUT5, solute carrier family 2 member 5; KHK, ketohexokinase; G6PD, Glucose-6-phosphate dehydrogenase; PPP, pentose phosphate pathway; SGs, stress granules; NUPR1, nuclear protein 1.

Pancreatic cancer cells are capable of specifically uptaking fructose via GLUT5. During metabolic reprogramming, fructose exerts synergistic regulatory effects on cell survival and proliferation through the PPP, lipid synthesis, and AMPK/mTORC1 signaling pathways (26–28), conferring metabolic plasticity to tumor cells to adapt to nutrient-deprived microenvironments (Figure 2).

The PPP is a critical intracellular metabolic route, broadly divided into oxidative and non-oxidative branches. The oxidative branch utilizes glucose-6-phosphate (G6P) as a substrate, catalyzed by enzymes such as glucose-6-phosphate dehydrogenase (G6PD), to generate NADPH and 5-phosphoribose (R-5-P). These products are essential for providing reducing power and precursors for nucleic acid synthesis. The non-oxidative branch, through the catalytic action of transketolase (TKT) and transaldolase (TAL), facilitates the interconversion of pentose sugars and glycolytic intermediates, thereby maintaining cellular carbon backbone balance and metabolic flux stability (29). TKT, with thiamine pyrophosphate as a cofactor, catalyzes the transfer of a keto group from a ketose to an aldose, shortening or elongating carbohydrate carbon chains. TAL catalyzes the transfer of an aldehyde group between aldoses and serves as a crucial enzyme linking the PPP’s non-oxidative branch to glycolysis. Together, these enzymes form the core catalytic system of the non-oxidative PPP, ensuring metabolic crosstalk between pentose metabolism and glycolysis (30). Fructose enters the PPP via fructose-6-phosphate (F6P). F6P can be directly phosphorylated from fructose by hexokinase (HK) or generated through gluconeogenesis. Subsequently, phosphoglucose isomerase converts F6P to G6P, allowing it to enter both the oxidative and non-oxidative branches of the PPP, providing R-5-P and xylulose-5-phosphate (Xu-5-P) as key substrates. On the one hand, the carbon skeletons derived from fructose metabolism are converted to Xu-5-P by TKT. Xu-5-P, a key intermediate in the non-oxidative PPP, can be further isomerized to R-5-P or epimerized to ribulose-5-phosphate, replenishing the PPP’s pentose pool (31). On the other hand, F6P can be directly converted to R-5-P through continuous catalysis by TAL and TKT, serving as a direct source for PPP’s nucleic acid precursor supply (32). Research by Jiao’s team at Lanzhou University in 2025 confirmed that KRASG12D mutations can activate the Rb/E2F1/BE2T signaling axis, promoting p53 degradation and thereby relieving its inhibition on G6PD. This establishes a positive feedback loop that enhances PPP metabolic activity, a mechanism identified as a key driver of malignant progression and targeted therapy resistance in KRAS-mutated pancreatic ductal adenocarcinoma (PDAC) (33). The generated R-5-P provides critical precursors for nucleic acid synthesis, supporting the rapid proliferation of pancreatic cancer cells. NADPH, in turn, maintains intracellular redox homeostasis, mitigating oxidative stress damage and ensuring cell survival (34). Furthermore, as the rate-limiting enzyme of the PPP’s oxidative branch, enhanced G6PD activity can increase NADPH production efficiency, preserving glutathione in its reduced state and protecting tumor cells from oxidative stress and chemotherapeutic drug damage. This protective mechanism has been extensively validated in pancreatic cancer chemoresistance (35). This entire process provides essential material synthesis and stress protection support for the “fructose metabolism-driven adaptive survival axis,” representing a critical metabolic node for the axis’s functional implementation.

Another metabolic pathway through which fructose enhances the invasiveness of pancreatic ductal adenocarcinoma is lipid synthesis, with fructose metabolism providing the necessary carbon sources and energy support for this process (26, 36). On the one hand, acetyl-CoA produced from fructose metabolism directly serves as the starting substrate for fatty acid synthesis, promoting the synthesis of cell membrane components such as phosphatidylcholine and maintaining the membrane structural integrity required for tumor cell proliferation (37). On the other hand, NADPH generated by enhanced PPP activity through fructose metabolism provides coenzymes for the reduction reactions in fatty acid synthesis, driving the sustained activation of lipid synthesis pathways (38). Furthermore, high fructose intake can be metabolized in the liver to produce lysophosphatidylcholine (LPC). Transported via the bloodstream to the pancreatic cancer microenvironment, LPC is taken up by cancer cells and further converted into phosphatidylcholine, thereby amplifying the supportive role of lipid synthesis in cell proliferation (39). This mechanism has been validated across multiple tumor models (40, 41). Inhibiting key enzymes in hepatic fructose metabolism significantly reduces serum LPC levels, thereby suppressing pancreatic cancer cell proliferation (36), including in PDAC-related models and KRAS-driven systems. This upregulated lipid synthesis not only fulfills the structural requirements of rapid tumor proliferation but also augments the invasiveness of tumor cells via the remodeling of membrane components.

### 3.3 Signal-stress output layer: fructose induces autophagy inhibition in pancreatic cancer cells via the AMPK/mTORC1 signaling pathway

Fructose can balance autophagy and proliferation by regulating the AMPK/mTORC1 signaling pathway. In glucose-deprived tumor microenvironments, GLUT5-mediated fructose uptake and metabolism suppress AMPK activity, thereby activating the mTORC1 signaling pathway (25). This subsequently activates the downstream kinase p70S6 kinase (p70S6K), with more pronounced activation observed in cells overexpressing Glut5.

Clinical studies confirm that GLUT5 is highly expressed in PDAC tissues, and its fructose-mediated AMPK/mTORC1 regulatory axis is closely associated with tumor progression, serving as an independent biomarker for disease progression in PDAC patients (25). Activation of mTORC1 significantly suppresses autophagy-mediated cell death in pancreatic cancer cells while promoting protein synthesis and cell cycle progression, thereby maintaining cellular survival and proliferative capacity (42). Concurrently, Cui et al. (21) found that in fructose-containing media, the mTORC1 inhibitor RAD001 and AMPK activator Phen significantly counteracted fructose’s inhibitory effect on autophagy. Furthermore, adding 2,5-AM or fructose degradation inhibitors to fructose-containing media markedly increased glucose-starvation-induced autophagy. Collectively, these findings indicate that fructose serves as a critical metabolic buffer in PDAC, effectively countering starvation-induced autophagic stress. This signaling regulatory mechanism constitutes the core defense strategy of the “fructose metabolism-driven adaptive survival axis” against nutritional deprivation stress (43). Marked tumor decalcification and sparse vasculature in PDAC tissues exacerbate nutrient deprivation, making enhanced utilization of inorganic carbon sources and activation of alternative metabolic pathways critical for PDAC progression (44).

Furthermore, multiple studies demonstrate that fructose enhances PDAC cell migration and invasion *in vitro*, with high fructose promoting metastatic potential in advanced pancreatic cancer (45). Reveal by Hsieh et al. revealed that an invasive ABCG2-positive cell subpopulation achieves selective proliferation under fructose-substituted culture conditions. They elucidated the critical role of α 2,6-sialyltransferase (ST6Gal1) in fructose-responsive regulation, enhancing the invasiveness of pancreatic ductal adenocarcinoma cells (45). Suggesting ST6Gal1 as a potential therapeutic target for pancreatic cancer. This finding further expands the functional scope of the “fructose metabolism-driven adaptive survival axis,” demonstrating that this axis not only regulates tumor cell survival and proliferation but also profoundly contributes to the intensification of malignant phenotypes and the elevation of metastatic potential.

## 4 Synergistic adaptation mechanisms of fructose metabolism with oxidative stress and stress granules

### 4.1 Bidirectional regulation of cellular redox homeostasis by fructose metabolism

Fructose metabolism bidirectionally regulates cellular redox balance through multiple pathways. It induces oxidative stress through metabolic enzymatic reactions and mitochondrial dysfunction while exerting protective effects by activating NADPH production and antioxidant signaling pathways. The equilibrium of this bidirectional effect depends on fructose concentration, metabolic state, and cell type. This holds significant pathological relevance in tumors and metabolic diseases and represents one of the core features enabling the “fructose metabolism-driven adaptive survival axis” to flexibly respond to microenvironmental changes (46). Within this dual-action framework, fructose can generate reactive oxygen species (ROS) via non-enzymatic glycation (fructation) while simultaneously activating antioxidant defenses during acute stress. (1) Previous research has identified the role of the polyol pathway in NADH/NAD + redox imbalance stress and diabetic oxidative stress. Under diabetic conditions, excessive polyol pathway activation reduces the NADPH/NADP ratio, inhibits glutathione reductase (GSR), and leads to ROS accumulation. In physiological states, moderate fructose metabolism generates NADPH via the pentose phosphate pathway (PPP), replenishing the antioxidant system. This underscores that NADPH balance is a central node in bidirectional regulation (47). (2) Studies demonstrate that metabolic flux also determines redox directionality, with fructose metabolism rate and cellular energy status jointly regulating the balance between ROS production and clearance. On the one hand, KHK-C mediated metabolism consumes ATP, activating AMPK and promoting mitochondrial ROS generation. On the other hand, F-1-P produced during fructose metabolism activates the transcription factor ChREBP, which upregulates G6PD (the rate-limiting enzyme of PPP) and increases NADPH supply (48). Crucially, in the tumor microenvironment, Fructose metabolic reprogramming shifts redox balance toward a pro-cancer phenotype (49). (3) Fructose exhibits bidirectional regulation of redox status across different cell types. Recent studies reveal differential effects of fructose metabolism on redox homeostasis in liver, gut, and adipose tissues. In hepatocytes, chronic high-dose fructose intake induces lipid peroxidation and mitochondrial dysfunction, whereas short-term low-dose fructose activates the AMPK-SIRT1 axis, enhancing mitochondrial antioxidant capacity. In intestinal cells, sugar promotes short-chain fatty acid production by gut microbiota, enhancing GSH synthesis in intestinal epithelial cells. Simultaneously, high fructose induces intestinal barrier damage, and endotoxins (LPS) entering the bloodstream exacerbate systemic oxidative stress (50). Similarly, in adipose tissue, fructose metabolism increases ROS in adipocytes, promoting inflammation; however, it also enhances fat storage through PPARγ activation, thereby alleviating metabolic stress (51). Thus, fructose metabolism bidirectionally regulates cellular redox homeostasis through concentration levels, metabolic states, and cellular type differences. This dynamic regulatory capacity enables the “fructose metabolism-driven adaptive survival axis” to maintain tumor cell survival advantages within complex microenvironments.

### 4.2 Mechanism of fructose-induced stress granule formation

Stress conditions within the tumor microenvironment, such as hypoxia and nutrient deficiency, can trigger oxidative stress responses. Stress granules (SGs), dynamic, membrane-less organelles formed by cells under stress, are primarily assembled through liquid-liquid phase separation (LLPS). They consist of untranslated mRNA, RNA-binding proteins (e.g., G3BP1, TIA-1, FUS), and a small number of translation initiation factors (e.g., eIF2α, eIF4F complex) (52, 53). Their core functions include protecting mRNA from degradation, temporarily inhibiting cap-dependent protein translation to conserve cellular energy, and serving as a signal transduction hub that integrates metabolic stress with cellular response signaling (54, 55). The formation of SGs is crucial for the “fructoses metabolism-driven adaptive survival axis” to couple metabolic stress with signal response (56). Researcher Ryoo H. D. and his team discovered that fructose activates eIF2α phosphorylation through three pathways: This phosphorylation process inhibits translation initiation, promotes release of untranslated mRNA, and subsequently drives RNA-binding protein (RBP) aggregation, establishing foundational conditions for SG formation (57). This process constitutes the critical first step in fructose’s conversion of metabolic signals into stress responses, providing the initiation conditions for signaling within the “adaptive survival axis.”

Regarding core components for SGs assembly, another study confirmed that methylglyoxal (MG) produced during fructose metabolism modifies low-complexity domains (LCDs) in RBPs. Furthermore, fructose-induced oxidative stress enhances the phase separation capacity of RBPs. These dual effects collectively promote the formation of liquid-like aggregates—the core components of SG assembly—by RBPs such as G3BP1, TIA-1, and FUS (53). This discovery reveals the direct regulatory role of fructose metabolites in SG assembly, demonstrating the tight coupling between metabolism and structural formation within the “fructose-driven adaptive survival axis.” Notably, fructose metabolism via fructokinase rapidly depletes intracellular ATP, lowering the ATP/ADP ratio. Concurrently, fructose inhibits mitochondrial function, reducing ATP production. This energy shortage activates the AMPK signaling pathway, suppressing mTORC1 activity and ultimately promoting the formation of energy-deficiency-induced SGs (eSGs) (54). This mechanism further strengthens the link between fructose metabolism and SG formation, enabling the “adaptive survival axis” to rapidly initiate stress defense through energy sensing (9, 21).

Recent studies have further revealed that SGs actively sequester mRNAs associated with fructose metabolism (e.g., GLUT5, KHK, G6PD), forming negative feedback loops by regulating the expression of these key metabolic enzymes to modulate fructose metabolic rates. simultaneously, this mRNA regulation influences cellular metabolic reprogramming, indirectly governing the dynamic equilibrium of SGs (55). This negative feedback mechanism endows the “fructose metabolism-driven adaptive survival axis” with self-regulatory capacity, ensuring dynamic balance between metabolism and stress responses.

In summary, fructose establishes the foundational conditions for SG formation by activating eIF2α phosphorylation to promote RBP aggregation. Subsequently, the MG produced from its metabolism modifies the LCD of RBPs and enhances their phase separation capacity through induced oxidative stress, forming the core assembly components of SGs. Finally, fructose metabolism consumes ATP; the resulting ATP deficiency activates the AMPK signaling pathway, which inhibits mTORC1 activity and leads to SG formation. These convergent mechanisms position fructose as a potent upstream regulator of stress granule assembly through metabolic, oxidative, and translational stress signaling. The formation of SGs provides the critical structural support for the “fructose metabolism-driven adaptive survival axis” to achieve stress tolerance.

### 4.3 SGs as metabolic-signaling coupling platforms and their relationship with fructose metabolism-induced pancreatic cancer

Methylglyoxal (MG) produced by fructose metabolism modifies the SGs core protein (G3BP1), enhancing its phase separation capacity and stabilizing SGs. The mTORC1 pathway influences stress granule formation by regulating translation. Inhibition of the mTOR pathway suppresses cap-dependent protein translation, leading to accumulation of SG-associated protein TIA-1/TIAR-dependent 5’TOP mRNA within SGs. Conversely, stress granules modulate cellular translation by regulating eIF4F complex assembly, thereby inversely controlling mTORC1 activity and cell growth (56). Experiments confirm that knocking down G3BP1 (a core stress granule component) inhibits SG formation in pancreatic cancer cells, reduces cellular resistance to oxidative stress, and suppresses tumor growth *in vivo*. This suggests the mTORC1-stress granule axis may represent a critical branch of fructose-regulated pancreatic cancer progression (58, 59). This axis’s tight interaction with fructose metabolism constitutes the core signaling network of the “fructose metabolism-driven adaptive survival axis.” Compared to traditional pathways: SGs achieve spatiotemporal co-localization of signaling molecules through phase separation, amplifying fructose-induced mTOR/AMPK signaling differences to regulate more persistent metabolic reprogramming (60). This unique signal amplification mechanism enables the “adaptive survival axis” to initiate potent adaptive responses even under low fructose concentrations or weak stress signals, providing sustained survival advantages for tumor cells.

**FIGURE 3:**
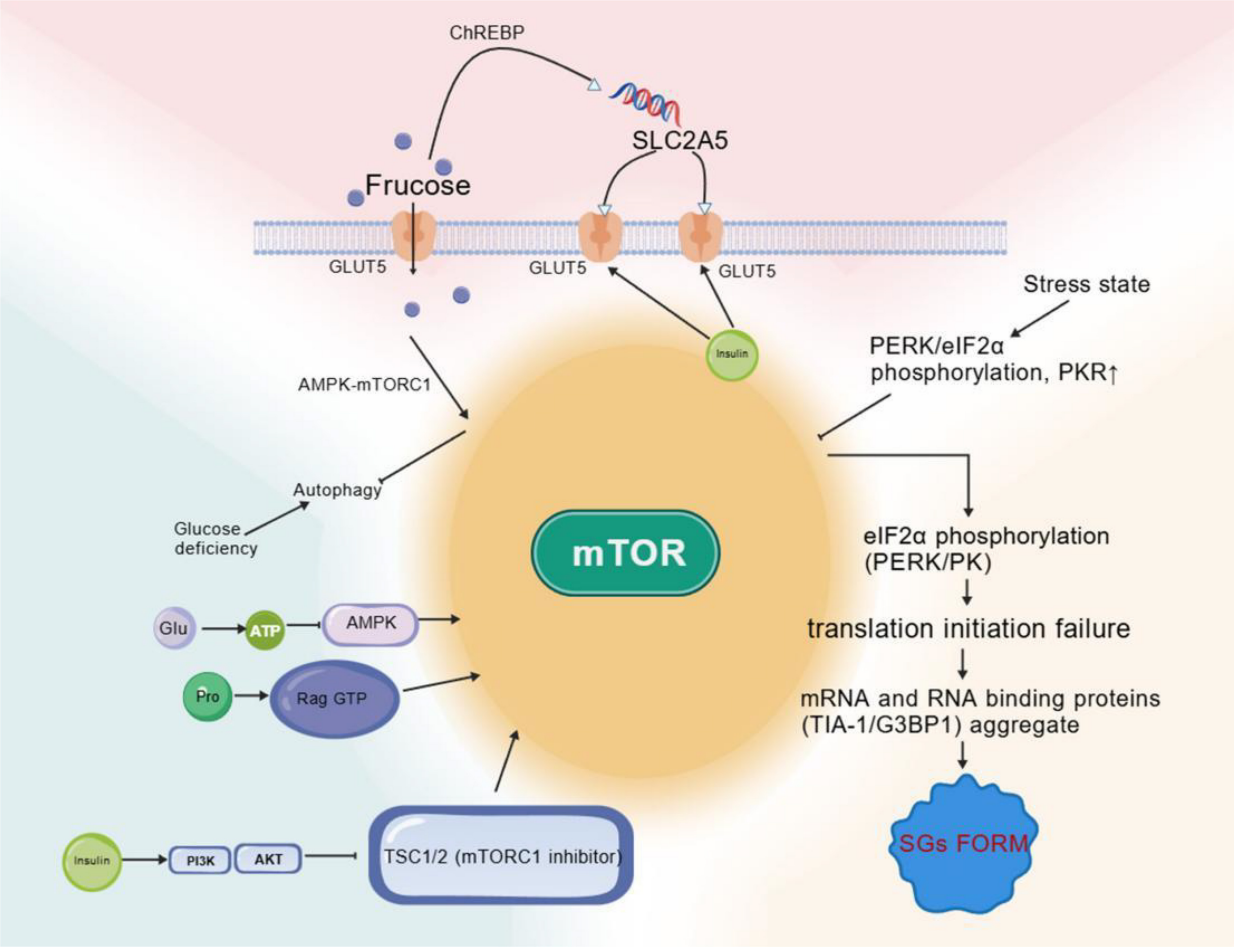
Mechanism of the mammalian target of rapamycin complex 1 (mTORC1)-stress granule signaling axis in regulating pancreatic cancer cell proliferation via fructose metabolism. Upon cellular uptake by pancreatic cancer cells via Glut5, fructose suppresses Activated Protein Kinase (AMPK) activation and promotes the mTORC1 pathway. The mTORC1 pathway, by modulating translation, drives the formation of stress granules (SGs). SGs, in turn, can inversely regulate cellular translation and growth through the modulation of eIF4F complex assembly. Concurrently, the stress granule core component G3BP1 interacts with nuclear protein 1 (NUPR1) to facilitate liquid-liquid phase separation (LLP), thereby enhancing cellular stress resistance. These mechanisms collectively promote the proliferation and progression of pancreatic cancer cells.

Within the tumor microenvironment, SGs mediate metabolic crosstalk between pancreatic cancer cells and stellate cells, promoting fructose-lactate shuttling and enhancing tumor invasiveness. Notably, KRAS G12D mutation enhances pancreatic cancer cell fructose uptake and metabolism by upregulating GLUT5 and KHK-C expression. Mutated KRAS promotes nuclear translocation of stress-induced nuclear protein 1 (NUPR1), which directly binds to SGs core proteins, enhancing phase separation efficiency and forming a KRAS-NUPR1-SGs oncogenic complex. This synergistic effect enables pancreatic cancer to maintain SG homeostasis through fructose metabolism even in low-glucose microenvironments, conferring a survival advantage (61). Stress-induced nuclear protein 1 (NUPR1), an intrinsically disordered protein overexpressed in multiple tumors (62), interacts with the stress granule core component G3BP1 to induce liquid-liquid phase separation and promote SG formation (58). Studies confirm that NUPR1 inhibitors block SGs formation and pancreatic intraepithelial neoplasia (PanIN) progression, with their-induced pancreatic cancer cell apoptosis dependent on KRAS expression, suggesting NUPR1 may be a key stress-regulatory molecule in fructose metabolism (63). This establishes a KRAS-driven, fructose-driven stress adaptation circuit centered on NUPR1-mediated phase separation (Figure 3).

## 5 Therapeutic implications and intervention strategies

### 5.1 Targeting fructose uptake

Based on the aforementioned studies, the Glut5-AMPK/mTORC1 signaling pathway may represent a potential therapeutic target for PDAC from a treatment perspective. Clinical research has demonstrated that blocking fructose uptake improves leukemia phenotypes and enhances the efficacy of the antileukemic drug Ara-C (Clinical trial numbers: NCT00001224, NCT03476793) (11). Angiogenesis is a critical step in tumor growth and metastasis. However, the unique characteristics of pancreatic cancer vasculature—including low microvascular density, immature structure, and functional leakage—have led to disappointing outcomes in clinical trials using anti-angiogenic drugs (64). Fructose analog 2,5-didehydro-D-mannitol (2,5-AM) was first validated by Wang et al. (65) to attenuate tumor angiogenesis when used in combination with fruitinib (Clinical Trials Nos: NCT02314169, NCT03626348), offering a novel therapeutic strategy for more precise pancreatic cancer treatment. The research involving 2,5-AM included preclinical *in vitro* experiments using the pancreatic cancer cell lines PANC-1 and MiaPaCa-2, and *in vivo* experiments utilizing a xenograft model of pancreatic cancer in nude mice (BALB/c-nu/nu). Although no direct GLUT5 inhibitors have been developed for pancreatic cancer treatment to date, research by Włodarczyk et al. investigating the inhibition of fructose uptake to suppress colorectal cancer cell growth has shown promising results. Their preclinical *in vitro* studies, utilizing colorectal cancer cell lines HT-29, LoVo, and Caco-2, demonstrated that the GLUT5 inhibitor MSNBA (10 μM) significantly reduced colorectal cancer cell viability with minimal impact on normal colonic epithelial cell survival (66). Targeting key fructose uptake molecules like GLUT5 offers a direct method to interrupt the substrate supply of the “fructose metabolism-driven adaptive survival axis,” thereby dismantling tumor metabolic adaptability at its source and presenting a precise therapeutic target for pancreatic cancer.

### 5.2 Targeting metabolic enzymes

(1) Targeting fructose kinase (KHK). Fukuda et al. reported results from the first-in-human clinical trial of LY3522348 (Clinical trial numbers: NCT04752153), a novel oral KHK inhibitor developed by Eli Lilly. LY3522348 is a dual-subtype KHK inhibitor (simultaneously inhibiting KHK-C and KHK-A) exhibiting favorable safety and pharmacokinetic profiles. It significantly reduced serum fructose-1-phosphate (F1P) levels following both single and multiple doses, confirming its effective inhibition of KHK enzyme activity. This provides clinical safety evidence for utilizing KHK inhibitors in treating metabolic-related diseases, including pancreatic cancer (67). Inhibiting KHK blocks the key rate-limiting step in fructose metabolism, preventing the initiation of metabolic reprogramming along the “adaptive survival axis” and thereby suppressing tumor-dependent metabolic adaptation. Notably, the clinical trials for LY3522348 did not progress to the final stage, which has become a significant point of contention regarding the clinical translation of KHK inhibitors. The specific official reason for the trial termination remains unclear, but it is speculated to be related to unexpected adverse reactions in subsequent trials, failure to meet predefined clinical endpoints, or adjustments in the pharmaceutical company’s R&D strategic layout. The discontinuation of this drug’s development offers important insights for future research on KHK inhibitors in the pancreatic cancer field. The target inhibition activity and short-term safety validated in its first-in-human trial provide critical clinical evidence for the development of similar drugs, confirming the feasibility of targeting KHK to interfere with fructose metabolism. Simultaneously, it suggests that subsequent R&D efforts should focus on pancreatic cancer specificity, developing KHK-C subtype selective inhibitors to reduce off-target side effects, incorporating pancreatic cancer-specific patient populations into clinical trials for precise validation of drug efficacy and safety, and balancing target inhibition efficiency with *in vivo* metabolic homeostasis to clear obstacles for the clinical translation of this class of drugs.

(2) Targeting Hexokinase 2 (HK2). Preclinical studies have investigated drugs exemplified by 3-bromopyruvate (3-BP) and lonidamine. The experiments for 3-BP utilized the pancreatic cancer cell lines PANC-1, BxPC-3, and AsPC-1, while studies employed xenograft models in nude (BALB/c-nu/nu) and SCID mice. For lonidamine, studies used MiaPaCa-2 and PANC-1 pancreatic cancer cell lines, with experiments also employing xenograft models in nude (NOD/SCID) mice. Both agents inhibit glucose phosphorylation, a critical initial step in glycolysis, thereby impeding glycolytic metabolism in tumor cells, reducing energy production, and concurrently inhibiting the binding of HK2 to the outer mitochondrial membrane, thereby relieving its apoptotic suppression. Garcia et al. (68) elucidated the characteristic high expression of HK2 in pancreatic cancer and its mechanism of action, confirming the significant anti-tumor efficacy of its inhibitors, 3-BP and lonidamine, in animal models. HK2 inhibitors can synergistically block the cross-talk between glucose and fructose metabolism, thereby potentiating the inhibition of the “fructose metabolism-driven adaptive survival axis.”

(3) Targeting Glucose-6-Phosphate Dehydrogenase (G6PD). Representative drugs include 6-aminonicotinamide (6-AN) and dehydroepiandrosterone (DHEA), both of which have undergone preclinical investigation. *In vitro* studies utilizing 6-AN employed pancreatic cancer cell lines PANC-1 and MiaPaCa-2 (chemo-resistant strains), while *in vivo* experiments utilized a chemo-resistant pancreatic cancer xenograft model in nude mice (BALB/c-nu/nu). For DHEA, *in vitro* studies used pancreatic cancer cell lines AsPC-1 and BxPC-3, and *in vivo* experiments employed an orthotopic pancreatic cancer transplantation model in nude mice. Both agents primarily function by inhibiting the activity of key enzymes in the PPP, thereby reducing nicotinamide adenine dinucleotide phosphate (NADPH) generation and enhancing the susceptibility of tumor cells to oxidative stress. Research by Deepak et al. (69) revealed aberrant activation of glucose-6-phosphate dehydrogenase (G6PD) in chemo-resistant pancreatic cancer, demonstrating that the combination of 6-AN with chemotherapeutic agents significantly augments therapeutic efficacy. G6PD, as a pivotal enzyme regulating redox homeostasis within the “adaptive survival axis,” its inhibition disrupts the tumor’s stress-protective mechanisms and elevates therapeutic sensitivity.

(4) Targeting Aldolase B (ALDOB): Currently, small-molecule inhibitors targeting this enzyme remain in the developmental stage. All relevant studies were preclinical *in vitro* experiments utilizing the PANC-1 and MiaPaCa-2 pancreatic cancer cell lines, the latter of which is a chemoresistant variant. Their mechanism involves inhibiting F-1-P degradation to block the abnormally activated fructose metabolic bypass in pancreatic cancer. However, Li et al. (70). identified a GLUT1/ALDOB/G6PD metabolic axis in chemotherapy-resistant pancreatic cancer, where elevated ALDOB expression establishes a fructose metabolic bypass, providing rationale for ALDOB-targeting drug development. These drugs primarily target fructose-dependent pancreatic cancers and are often used synergistically with GLUT1 inhibitors. This dual blockade of tumor cells’ metabolic utilization of fructose and glucose precisely targets tumors with specific metabolic phenotypes (70). Targeting ALDOB can disrupt the metabolic bypass of the “adaptive survival axis,” preventing tumors from evading treatment through metabolic reprogramming.

### 5.3 Combined therapeutic strategies

Combined therapies targeting fructose metabolism and stress granules offer a novel treatment paradigm for pancreatic cancer, particularly for patients with KRAS mutations, high GLUT5 expression, and obesity-associated PDAC. For SGs, clinically, NUPR1 inhibitors such as ZZW-115/ZW-115 are currently available, with related research in the preclinical stage. *In vitro* experiments utilized pancreatic cancer cell lines PANC-1 and MiaPaCa-2 (KRASG12D mutant strains), while *in vivo* experiments employed a xenograft model of KRASG12D mutant pancreatic cancer in nude mice (BALB/c-nu/nu). These inhibitors compete with imported proteins to bind to the NLS region of NUPR1, thereby inhibiting its nuclear translocation (71). This reduces the SUMOylation-dependent function of key proteins involved in the DNA damage response, sensitizing cancer cells to genotoxic drugs. Concurrently, blocking NUPR1-driven LLPS inhibits SG formation while inducing ferroptosis and necrotic apoptosis (61). Fonteneau et al. identified a previously unknown SG regulator and component, serine/arginine protein kinase 2 (SRPK2), as a specific determinant of SG formation in obesity-associated PDAC. SRPK2-mediated SG formation in obesity-associated PDAC is driven by overactivation of the IGF1/PI3K/mTOR/S6K1 pathway, a target potentially inhibited by the S6K1 inhibitor PF-4708671 (72). Preclinical investigations utilizing the S6K1 inhibitor PF-4708671 involved *in vitro* studies with pancreatic cancer cell lines PANC-1 and MiaPaCa-2, recognized models of obesity-associated PDAC. *In vivo* experiments employed an orthotopic xenograft model in diet-induced obese (DIO) nude mice. The mechanism of action involves the inhibition of the IGF1/PI3K/mTOR/S6K1 signaling axis.

Furthermore, preclinical studies investigating small molecule inhibitors targeting G3BP1, such as C93 and ST101, have utilized the PANC-1 and AsPC-1 pancreatic cancer cell lines for *in vitro* experiments and an orthotopic xenograft model in nude mice for *in vivo* studies. These models are amenable to investigating the functions of SG (73). It follows that NUPR1 inhibitors/S6K1 inhibitors combined with fructose metabolism inhibitors would simultaneously block energy supply (fructose metabolism) and stress adaptation (SG formation), inducing synthetic lethality. This combined strategy comprehensively disrupts the core function of the “fructose metabolism-driven adaptive survival axis,” achieving synergistic antitumor effects. Future investigations should carry out fructose gradient exposure experiments and large-scale population cohort studies to delineate dose-response relationships and establish intake thresholds.

### 5.4 Potential of precision nutrition and lifestyle interventions

Fructose intake is significantly associated with pancreatic cancer risk, exerting its carcinogenic effects through mechanisms including metabolic reprogramming, activation of the NUPR1-stress granule pathway, and gut microbiome dysbiosis. Precision nutrition and lifestyle interventions, by integrating genetic, metabolic, and microbiome data, enable personalized fructose restriction protocols tailored to different risk groups. Combined with exercise and weight management, these approaches can effectively reduce pancreatic cancer risk, particularly in high-risk individuals such as those with KRAS mutations. With the development of targeted strategies like NUPR1 inhibitors, a comprehensive pancreatic cancer prevention system integrating “precision stratification-targeted intervention-early screening” is anticipated in the future, offering new avenues to reduce the incidence of this lethal tumor (Table 2). This approach can suppress the activation of the “fructose metabolism-driven adaptive survival axis” at its source, complementing targeted therapies to establish a comprehensive prevention and control system.

**TABLE 2:** Fructose intake control measures for populations at different risk of pancreatic cancer.

No.	Population	Symptoms	Control measures	Recommendations	References
1	High-risk population	KRAS mutation, positive family history	Strict fructose restriction, avoiding sugar-sweetened beverages, processed foods, and high-fructose fruits (e.g., mangoes, lychees); low-dose ZZW-115 combined with dietary intervention to block stress granule formation.	Supplementing with omega-3 fatty acids (from deep-sea fish), curcumin, and green tea to mitigate pancreatic inflammation. Engaging in at least 150 min of moderate-intensity aerobic exercise weekly can reduce insulin resistance and stress levels.	(15, 49, 61, 71)
2	Medium-risk population	Metabolic syndrome, obesity	Moderate fructose restriction, prioritizing low-fructose fruits (e.g., blueberries, strawberries) and whole fruits (avoiding juices); optimization of the gut microbiome, such as supplementation with Bifidobacterium and other lactic acid bacterial probiotics; increased intake of soluble dietary fibers (e.g., inulin, oats).	To maintain a Body Mass Index (BMI) within the healthy range of 18.5–24.9, a calorie-restricted diet combined with strength training is advised. Monitoring blood glucose, insulin, and uric acid levels every 6 months will allow for dynamic adjustments to the intervention plan.	(15, 72)
3	General population	Primary prevention	Judicious fructose intake, limiting added sugars.	Prioritize whole-food sources of fructose and engage in at least 150 min of physical activity per week. Individuals over 40 should undergo annual pancreatic ultrasound examinations, with high-risk groups additionally monitored via CA19-9 testing.	(4, 9, 15)

## 6 Discussion

This review systematically integrates the core mechanisms by which fructose metabolism regulates pancreatic cancer progression, innovatively proposing the conceptual framework of the “fructose metabolic adaptive survival axis.” It elucidates that fructose metabolism functions not merely as an alternative carbon source, but as a key regulatory strategy enabling pancreatic cancer to gain survival advantages in nutrient-deprived, oxidative stress microenvironments—spanning the entire chain from metabolic input and reprogramming to signal output. Compared to previous reviews, this study achieves significant breakthroughs in three key aspects: depth of mechanism integration, analysis of heterogeneity, and translational orientation. These are explored in depth below through core dimensions.

### 6.1 Dose- and spatiotemporal-dependent effects of fructose

The core innovation lies in the first systematic integration of expression regulation mechanisms for dual fructose sources. This study clarifies the synergistic interaction pattern between exogenous fructose (cross-organ transport via gut-liver-pancreas) and endogenous fructose (activated via the polyol pathway), filling gaps in prior reviews regarding fructose source heterogeneity and regulatory network exploration. Previous studies predominantly analyzed isolated fructose metabolic pathways and were confined to the broad conclusion that “high fructose promotes carcinogenesis,” failing to distinguish the regulatory effects of fructose dose, exposure duration, and source differences on pancreatic cancer progression. Through stratified analysis, this study reveals that fructose regulation of pancreatic cancer exhibits significant dual dependence on dose and spatiotemporal factors. This dual dependency forms the foundation for the flexible functioning of the “adaptive survival axis driven by fructose metabolism.”

Regarding dose dependency, fructose exhibits bidirectional regulation of redox homeostasis in pancreatic cancer cells. The equilibrium of this effect directly depends on fructose concentration, cellular metabolic state, and cell type differences. At physiological doses, naturally occurring fructose coexists with dietary fiber and is absorbed slowly. NADPH is primarily generated via the PPP, replenishing the cellular antioxidant system and maintaining redox balance (47). At pathological doses, however, fructose from added sugars overloads metabolic capacity. It activates the polyol pathway, induces mitochondrial dysfunction, and generates reactive oxygen species (ROS) through non-enzymatic glycation reactions, thereby inducing oxidative stress. Concurrently, it enhances fructose uptake by upregulating GLUT5 expression, forming a pro-cancer metabolic cycle (49). From a spatiotemporal perspective, short-term acute fructose exposure primarily regulates cellular autophagy balance by activating the AMPK/mTORC1 signaling pathway, while long-term chronic exposure drives sustained metabolic reprogramming activation. Concurrently, it induces abnormal upregulation of endogenous fructose synthesis pathways, providing stable metabolic support for tumor cells (18).

At the metabolic reprogramming level, this study further clarifies two core pathways by which fructose regulates pancreatic cancer. The first is the pentose phosphate pathway (PPP). After being metabolized into fructose-6-phosphate (F6P), fructose enters the non-oxidative branch of PPP via reactions mediated by transketolase (TKT) and transaldolase (TAL), yielding ribose-5-phosphate (R5P) and NADPH (32). where R5P provides a key precursor for nucleic acid synthesis, supporting rapid proliferation of pancreatic cancer cells, while NADPH maintains intracellular redox balance and enhances stress resistance (21). Furthermore, in KRAS-mutated pancreatic ductal adenocarcinoma (PDAC), this pathway can release p53 inhibition on G6PD through a UBE2T-mediated feedback mechanism, further increasing metabolic flux (33). The second pathway involves lipid synthesis: acetyl-CoA generated from fructose metabolism directly serves as a starting substrate for fatty acid synthesis, promoting the production of cell membrane components such as phosphatidylcholine (37). While NADPH generated via the pentose phosphate pathway (PPP) provides coenzymes for fatty acid synthesis reduction reactions. Additionally, lysophosphatidylcholine (LPC) produced from hepatic fructose metabolism can be transported to the pancreatic microenvironment, further amplifying lipid synthesis’s support for cell proliferation (38). Ultimately, this study corroborates that fructose, through GLUT5-mediated uptake and metabolism, inhibits AMPK activity and activates the mTORC1 signaling pathway. This not only significantly suppresses autophagy-mediated death in pancreatic cancer cells but also promotes protein synthesis and cell cycle progression (42). Concurrently, it enhances the *in vitro* migration and invasive capabilities of PDAC cells (45), forming a complete regulatory chain of “metabolic supply -signaling regulation-malignant phenotype” regulatory chain. This chain constitutes the core functional module of the “fructose metabolism-driven adaptive survival axis.”

### 6.2 Fructose promotes PDAC progression by inducing SGs formation

A key breakthrough in this study lies in focusing on cross-organ metabolic regulation between the liver and pancreas. We elucidated the regulatory pathway whereby fructose metabolism in the liver generates LPC, which is transported to the pancreatic microenvironment to enhance lipid synthesis. Simultaneously establishing an innovative “fructose metabolism-redox balance-stress granule (SG) formation” coupling network. This completes the full molecular mechanism chain of fructose regulating pancreatic cancer progression, filling a gap in previous reviews that inadequately explored the synergistic mechanisms between fructose metabolism and stress responses (9, 15, 74). Previous studies have predominantly analyzed either the fructose metabolic pathway or the stress-protective function of SGs in isolation, failing to reveal their synergistic pro-cancer effects. This study, through mechanistic integration, demonstrates that SGs serve as a metabolic-signaling coupling platform, playing a central mediating role in fructose-regulated pancreatic cancer progression. They constitute the key structural element enabling signal integration and stress tolerance within the “fructose metabolism-driven adaptive survival axis.”

This review delineates that fructose orchestrates SGs assembly via three functionally interconnected molecular pathways. First, it activates eIF2α phosphorylation, which in turn activates PERK kinase via endoplasmic reticulum stress, PKR kinase via oxidative stress, and GCN2 kinase via amino acid deprivation. This inhibits translation initiation and promotes untranslated mRNA release, laying the foundation for SGs formation (57). Second, fructose metabolism produces methylglyoxal (MG), which modifies low-complexity domains (LCDs) of RNA-binding proteins (RBPs). Concurrently, fructose-induced oxidative stress enhances the phase-separation capacity of RBPs, promoting the formation of liquid-phase aggregates by RBPs such as G3BP1, TIA-1, and FUS, which constitute the core assembly components of SG (53). Third, fructose rapidly depletes intracellular ATP via fructokinase metabolism, lowering the ATP/ADP ratio. Concurrently, it inhibits mitochondrial function to reduce ATP production, activates the AMPK signaling pathway, and suppresses mTORC1 activity, thereby promoting the formation of energy-deficiency-induced SG (eSG) (54). More critically, SGs form negative feedback loops by sequestering mRNA of key fructose metabolism enzymes like GLUT5, KHK, and G6PD, dynamically regulating fructose metabolism rates to achieve precise metabolic-stress response equilibrium (55). This negative feedback regulation endows the “adaptive survival axis” with self-stabilizing capacity, preventing metabolic overactivation or insufficient stress responses, thereby ensuring tumor cells maintain optimal survival states within complex microenvironments.

This study further elucidates the decisive regulatory role of molecular characteristics in determining fructose metabolic phenotypes, revealing a synergistic regulatory mechanism involving KRAS^12^ mutations, KHK gene polymorphisms, and androgen receptor (AR) expression levels: KRAS^12^ mutations enhance the synergistic oncogenic effects of fructose metabolism and SG formation by upregulating GLUT5 and KHK expression and inducing nuclear translocation of stress-induced nuclear protein 1 (NUPR1). Mutated KRAS also promotes NUPR1 interaction with G3BP1, a core SG protein, leading to liquid-liquid phase separation and increased SG stability (61). Selective expression of KHK-C isoforms directly determines fructose metabolic flux. Their expression is regulated by the transcription factor ChREBP and can be induced by dietary fructose, serving as a key switch for initiating fructose metabolism (49). High AR expression accelerates endogenous fructose synthesis via the polyol pathway, particularly under hypoxia and Warburg effect induction (37). Synchronous upregulation of aldose reductase (AR/AKR1B1) and sorbitol dehydrogenase (SDH/SORD) further enhances tumor cell metabolic flexibility (38). Based on these findings, the concept of the “fructose metabolic adaptive survival axis” was proposed. represents a core innovation by challenging the conventional view that “fructose serves only as an alternative carbon source.” It reveals that fructose metabolism constitutes an active survival strategy regulated by tumor cells. By reshaping metabolic plasticity and stress adaptation capacity, this pathway enhances pancreatic cancer’s tolerance to nutrient-deprived and oxidative stress microenvironments. This approach addresses a critical gap in existing research—the insufficient focus on individual heterogeneity—and provides robust theoretical support for precision interventions based on molecular subtyping.

### 6.3 Therapeutic approaches targeting the fructose metabolism adaptive survival axis and exposure risk intervention

Based on the “fructose metabolic adaptive survival axis” framework, this study innovatively proposes a synthetic lethal therapeutic strategy targeting fructose uptake/metabolic enzymes while blocking SG formation. This approach bridges the gap between basic research and clinical application. Compared to traditional chemotherapy and anti-angiogenic therapies, it better aligns with the pathophysiological characteristics of pancreatic cancer—specifically its “hypovascularity and nutrient deprivation”—offering enhanced targeting potential and clinical translation prospects. This represents the core distinction between our study and previous reviews, which often focused solely on mechanism summaries while lacking clinical translation orientation nutrient-deprived pathological characteristics, demonstrating superior targeting potential and clinical translational promise. This represents the core distinction between this study and previous reviews in translational orientation—earlier reviews often remained confined to mechanism summaries, lacking systematic integration of targeted therapeutic strategies. In contrast, this study constructs a complete translational chain linking “mechanism-target-intervention,” offering a novel pathway for precision treatment of pancreatic cancer.

At the targeted therapy level, this study identifies three core intervention targets and synergistic strategies. First, targeting fructose uptake: GLUT5, as a fructose-specific transporter, has demonstrated significant reduction in cancer cell survival with low toxicity to normal cells in colorectal cancer studies using its inhibitor MSNBA (66), showing potential for extension to pancreatic cancer treatment. Additionally, the fructose analog 2,5-anhydro-D-mannitol (2,5-AM) combined with fruitinib slows tumor angiogenesis, providing evidence for synergistic effects between fructose metabolism targeting and anti-angiogenic therapy (65). Second, targeting fructose metabolic enzymes: The KHK inhibitor LY3522348, as a dual-subtype inhibitor, has demonstrated favorable safety and KHK enzyme activity inhibition in initial human clinical trials, laying the foundation for its application in pancreatic cancer treatment (67). Additionally, glucose-6-phosphate dehydrogenase (G6PD) inhibitors such as 6-aminonicotinamide (6-AN) and hexokinase 2 (HK2) inhibitor 3-bromopyruvate (3-BP), and others can enhance tumor cell sensitivity to oxidative stress by blocking downstream pathways of fructose metabolism, making them particularly suitable for chemotherapy-resistant pancreatic cancer (68, 69). Third, targeting SGs formation: NUPR1 inhibitor ZZW-115 suppresses NUPR1 nuclear translocation, blocking its interaction with G3BP1 and preventing droplet formation (71). Simultaneously, serine/arginine protein kinase 2 (SRPK2) inhibitor PF-4708671 targets SGs formation in obesity-associated PDAC (72). When used synergistically with fructose metabolism inhibitors, these agents simultaneously disrupt tumor cell energy supply and stress adaptation capacity, achieving synthetic lethality. These targeted therapeutic strategies exert their effects on discrete functional modules of the “fructose metabolism-driven adaptive survival axis.” Used alone, they inhibit localized modules of the axis; combined, they comprehensively dismantle the axis’s overall function, yielding highly effective antitumor outcomes.

At the exposure risk intervention level, this study integrates fructose source heterogeneity and population risk variations to establish a three-dimensional prevention and control system: “risk stratification-targeted therapy-lifestyle intervention.” For high-risk populations (e.g., KRAS mutation carriers, positive family history), strict fructose restriction is recommended—avoiding added sugars and high-fructose fruits—combined with low-dose ZZW-115 to block SGs formation, alongside supplementation of omega-3 fatty acids and curcumin to suppress pancreatic inflammation (15, 49, 61, 71). For moderate-risk groups with metabolic syndrome or obesity, moderate fructose restriction is advised, prioritizing low-fructose fruits and whole foods. Gut microbiome optimization through probiotic and soluble fiber supplementation should be combined with BMI and metabolic marker control (15, 72). For the general population, primary pancreatic cancer prevention is achieved through intelligent fructose intake control, consistent exercise, and regular screening (4, 9, 15). This system integrates genetic profiling, metabolic status, and lifestyle factors to provide personalized intervention plans for different risk groups. By reducing fructose exposure to inhibit activation of the “fructose metabolism-driven adaptive survival axis,” it lowers pancreatic cancer risk at its source while synergizing with targeted therapies to improve patient prognosis. This comprehensive strategy of “precision stratification—early intervention—targeted therapy” strategy.

### 6.4 Limitations and future research

Although this study preliminarily establishes a theoretical framework for fructose metabolism regulation in pancreatic and long-term population data. Regulatory thresholds under different exposure conditions cancer, several limitations remain. (1) The dose- and spatiotemporal-dependent analysis of fructose lacks support from independent gradient exposure experiments and metabolic differences between natural fructose and added sugars require further quantification and validation. (2) Conclusions are partially derived from traditional animal models, with incomplete validation of the “fructose metabolism adaptive survival axis” in PDX or organoid models, limiting clinical extrapolation. (3) The mechanisms of metabolic crosstalk among cellular subpopulations in the tumor microenvironment remain under-explored, and core molecular hubs within the fructose metabolic network have not been identified through multi-omics integration; (4) Combined therapeutic strategies targeting fructose metabolism and SG formation remain in the preclinical stage, constrained by the absence of large-scale clinical trials and standardized molecular biomarker detection systems, hindering clinical translation.

Future research should: Conduct fructose gradient exposure experiments and large-scale population cohort studies to define dose-response relationships and intake thresholds; Develop clinically relevant composite models (e.g., fructose combined with metabolic comorbidities) and validate these metabolic axes using PDX and organoid models; Employ single-cell and multi-omics technologies to decipher intercellular metabolic dialogs and identify key regulatory nodes; Advance the clinical translation of combined treatment regimens, establish standardized biomarker detection systems, and ultimately provide novel support for precision interventions in pancreatic cancer.

## 7 Conclusion

Pancreatic ductal adenocarcinoma (PDAC) exhibits remarkable metabolic plasticity, enabling tumor cells to survive and proliferate under conditions of extreme nutrient deprivation and oxidative stress. This review systematically integrates the latest evidence confirming that fructose metabolism functions not merely as an alternative carbon source, but as a coordinated and actively regulated survival strategy in pancreatic cancer. At the pathway level, conceptualizing fructose metabolism as an adaptive survival axis for pancreatic cancer facilitates a reevaluation of holistic intervention strategies for prevention and treatment. Traditional metabolic targeting strategies often focus on single pathways or molecular nodes, whereas the metabolic-stress coupling network formed by fructose metabolism suggests that pancreatic cancer’s survival advantage stems from the coordinated operation of multi-level adaptive mechanisms. Based on this understanding, future intervention strategies may shift from single-point inhibition to systematic regulation across input layers (dietary and metabolic exposure control), signal integration layers (Glut5–AMPK/mTORC1 axis), and stress execution layers (stress granules and translational regulation). This provides a theoretical framework for developing more tolerable and precise metabolism-directed therapeutic approaches.

By integrating exogenous dietary fructose intake with endogenous fructose synthesis via the polyol pathway, we reveal a multi-tiered regulatory network. Fructose drives pentose phosphate pathway flux, lipid biosynthesis, and AMPK-mTORC1 signaling, thereby maintaining redox balance, suppressing excessive autophagy, and promoting malignant phenotypes. Notably, we highlight stress granules (SGs) as an underappreciated metabolic-signaling interface that converts fructose-induced redox and energy stress into adaptive regulatory responses, thereby enhancing tumor stress tolerance. Based on these convergent mechanisms, we propose the concept of a “fructose-driven adaptive survival axis” in PDAC, integrating metabolic reprogramming, stress responses, and oncogenic signaling pathways into a unified framework. This not only deepens our understanding of pancreatic cancer metabolism but also reveals exploitable therapeutic vulnerabilities. Targeting fructose uptake, key metabolic enzymes, or glycogen storage disease-associated regulators—either individually or in combination—offers a promising strategy to simultaneously disrupt tumor energy supply and stress adaptation. Furthermore, integrating metabolic targeting with patient stratification and precision nutritional interventions may open new avenues for preventing and treating KRAS-mutant, GLUT5-overexpressing, or obesity-associated PDAC. Collectively, this study provides a conceptual and translational foundation for rethinking fructose metabolism as a key determinant of pancreatic cancer progression and therapeutic resistance.
